# Neuromyelitis Optica Spectrum Disease with Positive Autoimmune Indices: A Case Report and Review of the Literature

**DOI:** 10.1155/2011/393568

**Published:** 2011-11-01

**Authors:** M. E. Evangelopoulos, G. Koutsis, E. Andreadou, C. Potagas, A. Dimirakopoulos, C. Sfagos

**Affiliations:** Department of Neurology, Eginition Hospital, University of Athens, 74 Vas. Sophias Avenue, 11528 Athens, Greece

## Abstract

A 45-year-old female suffering from severe thoracic pain was admitted to the emergency department of our hospital. Thorough clinical examination revealed paresis of the left lower limb and sensory deficit at the level of the Th4 vertebra. MRI of the thoracic spine demonstrated a lesion at the level of Th1–Th7. Despite initial improvement following i.v. corticosteroid administration, the patient's clinical status deteriorated, with recurrence of myelitis and extension of the lesion to Th12. She developed paraparesis, hyperreflexia and spasticity of both legs, symmetrical sensory deficit below Th4, and sphincter dysfunction. Differential diagnosis included infectious, metabolic, neoplastic/paraneoplastic, and ischemic causes as well as multiple sclerosis. NMO IgG was found positive and led to the diagnosis of longitudinal extensive transverse myelitis (LETM) in the NMO spectrum disorders. Administration of immunosuppressive therapy resulted in gradual improvement of the patient's clinical status and stabilization for five years. In the setting of LETM, patients with antiaquaporin 4 IgGs can present features of coexisting systemic involvement. A thorough differential diagnosis is required to guide appropriate therapy.

## 1. Introduction

Longitudinal extensive transverse myelitis (LETM) represents a heterogeneous syndrome caused by an inflammatory process of the grey and white matter of the spinal cord, resulting in neurologic deficits such as weakness, sensory loss, and autonomic dysfunction [1]. Although its etiology remains unknown, infectious/parainfectious, ischemic, and metabolic causes are included in the differential diagnosis. LETM may be associated not only with multiple sclerosis, neuromyelitis optica (NMO) but also with systemic autoimmune diseases. The syndrome has rarely been reported to occur as a first manifestation of systemic lupus erythematosus (SLE) or Sjogren's syndrome [2–4]. 

 Neuromyelitis optica (NMO or Devic's disease) is a serious, idiopathic demyelinating disease of the central nervous system (CNS) selectively attacking the spinal cord or optic nerves [5]. Although originally considered a variation of multiple sclerosis (MS), clinical, radiological, pathological, and especially immunological data (detection of NMO IgG antibody) have led to a novel definition of this clinical entity [6]. NMO spectrum disorders include either recurrent longitudinally extensive myelitis (more than a three-vertebral-segment spinal cord lesion seen in MRI) or recurrent optic neuritis. NMO IgG autoantibody recognizing the water channel aquaporin-4 represents a highly specific biomarker for NMO as well as for NMO spectrum disorders and is considered as an additional criterion supporting the diagnosis [7, 8].

Early differential diagnosis of NMO from MS and systemic autoimmune disorders is of vital importance as far as therapeutic intervention is concerned, since NMO has a poor prognosis and requires immunosuppressive and not immunomodulatory treatment.

## 2. Case Report

We present the case of a 45-year-old female complaining of severe thoracic pain. On admission, initial clinical examination revealed impaired sensation of light touch, pain, and sense of vibration at the left lower limb, extending to the level of Th4. 

**Figure 1:**
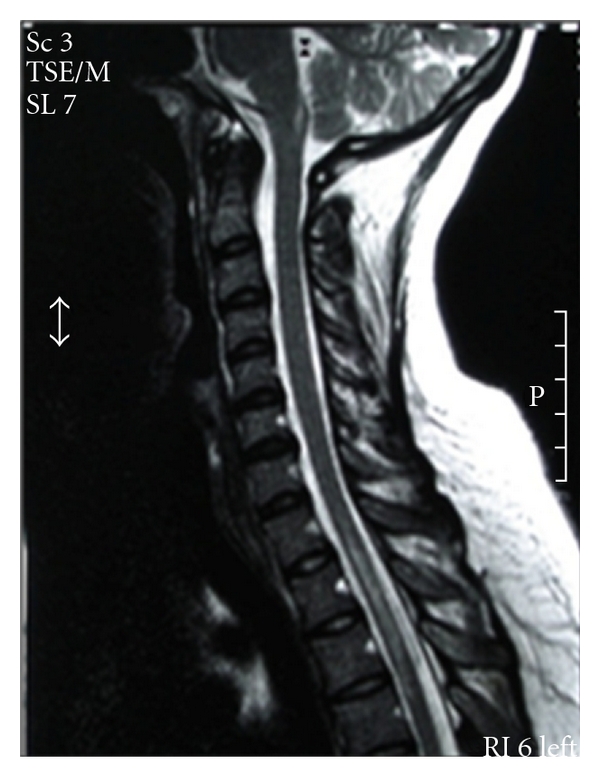
Sagital T2-MRI of the spinal cord showing an extensive enhancing thoracic lesion (Th1-Th3).

Thoracic spine MRI revealed a lesion in the spinal cord at the levels of Th1–Th7, while an enhancing signal was noted at the levels of Th1–Th3 (Figure 1). Brain MRI demonstrated no pathological findings; visual evoked potentials were normal. 

Testing for antibodies to HSV1, HSV2, VZV, CMV, EBV, HBV, HCV, HIV in serum and cerebrospinal fluid, as well as sarcoidosis and tumor markers in serum revealed no pathological values. Polymerase chain reaction in CSF for HSV1 and HSV2 was negative. 

Thoracic and abdominal CT scans were not indicative of any other pathology. Cerebrospinal fluid analysis demonstrated no lymphocytes, normal protein, and negative oligoclonal bands. Immunological tests revealed an ANA titer of 1 : 320, while tests for anti-ENA, anti-dsDNA, anticardiolipin, anti-*β*2GPI, lupus cells, and cryoglobulins were negative. 

An initial improvement in patient's clinical status was noted after administration of i.v. corticosteroids (1 gr methylprednisolone for 5 days). The patient presented with chest pain, paraparesis, hyperreflexia and spasticity of both legs, symmetrical sensory deficit below Th4, and sphincter dysfunction twenty days post conclusion of oral tapering of corticosteroid regimen. Clinical examination revealed bilateral Babinski signs, loss of sensitivity below Th4, and left lower extremity weakness with MRC grade 1/5 proximally and right leg MRC grade 2/5. Visual evoked potentials and CSF biochemical tests remained normal. However, MRI of the thoracic spine demonstrated an extension of the preexisting lesion (from Th1–Th3 to Th1–Th8) and thoracic CT scan revealed serositis with limited bilateral pleural effusion. After two weeks and while still hospitalized and receiving oral corticosteroid tapering, the patient developed recurrent thoracic myelitis and MRI demonstrated an extension of the lesion to the level of Th12. The blood count revealed leucopenia while the sedimentation rate was increased. Daily urinary protein loss also increased slightly, at 400 mg/day (0–200 mg/day). New immunological tests, performed at the Mayo Clinic, revealed the presence of NMO-IgG antibodies.

Since our patient presented several signs of autoimmune disease (positive ANAs, increased sedimentation rate, serositis, mild proteinuria, and gradual improvement following corticosteroids administration), initial differential diagnosis included autoimmune disorders (SLEs) as well as MS and NMO. After consulting with rheumatologists, it was decided that clinical and laboratory findings did not fulfill the diagnostic criteria for SLE. Taking into account the recurrent severe attacks of myelitis, the extensive spinal cord lesion (Th1–Th12), the absence of oligoclonal bands, the positive autoimmune clinical signs and indices, and the positive NMO-IgG antibodies, it was suggested that the patient was suffering from LETM of the NMO-SD type and immunosuppressive therapy was administered. 

Since i.v. cyclophosphamide is currently used to treat patients with lupus and life-threatening neurologic diseases, it was administered at a dose of 750 mg monthly for 6 months and then every 3 months for 8 times. Uromitexan was also administered with cyclophosphamide to prevent hemorrhagic cystitis Oral prednisone was slowly tapered to a daily dose of 10 mg. 

Our patient showed remarkable improvement two years after initial treatment. No motor deficit was detected while sense of light touch and pain were slightly decreased below Th4 and sense of vibration of the left leg remained impaired. However, three months later, while switching from i.v. cyclophosphamide to per os immunosuppressive treatment (azathioprine), she relapsed severely. Administration of corticosteroids i.v. and immunosuppressive treatment (cyclophosphamide 750 mg monthly for 6 months) led to complete patient's recovery. She remains in remission for three more years under treatment with rituximab (375 mg/m2 infused once per week for 4 weeks and then every two months) CD19 cell markers are routinely monitored.

## 3. Discussion

Longitudinal extensive transverse myelitis (LETM) may occur as an uncommon manifestation of SLE or other autoimmune diseases, appearing in only 2% of patients several years before the development of SLE [3, 4]. 

Differential diagnosis between the LETM that occurs in MS and that of autoimmune disorders such as SLE or NMO spectrum disorders is crucial, since different therapeutic interventions are required and interferon beta-1b treatment may induce flare-ups of both diseases [5, 6, 9, 10]. 

NMO IgG represents a highly specific diagnostic marker for NMO but also for NMO spectrum disorders (NMO-SDs), which include recurrent longitudinally extensive myelitis or recurrent optic neuritis [7]. 

NMO and NMO-SD have rarely been reported as a first manifestation of SLE and Sjogren's syndrome [2–4, 11, 12]. NMO and NMO-SD are rare pathological entities with serious neurological manifestations. Concerning their association with autoimmune disorders, recent literature data propose that seropositivity for NMO IgG in patients with SLE and Sjogren's syndrome is not an epiphenomenon, but rather these patients possibly suffer from two coexisting autoimmune disorders [5, 10, 13]. Pittock et al. demonstrated that NMO IgG help to distinguish LETM as an NMO-SD manifestation in patients with systemic autoimmune diseases. It is noteworthy that NMO IgG was only found in patients with SLE and coexisting LETM. On the other hand, non-organ-specific autoantibodies were frequently found in patients with NMO and NMO-SD [10]. Despite LETM and NMO IgG, during admittance our patient demonstrated clinical and biological signs of systemic involvement (serositis, leucopenia, increased ESR, and proteinuria). Nevertheless, she did not fulfill SLE diagnosis criteria. To our knowledge, there is only one more case of proteinuria in the setting of Devic's disease. However, this was a case of concomitant SLE [14]. In accordance with these findings, it is recently reported that the onset of NMOSD may precede the clinical and serological findings of systemic autoimmunity [15]. The difficulty of defining the underlying disease, especially at its onset, is clearly shown in our case.

The specificity of NMO IgG for NMOSD in patients with connective tissue diseases (CTD) was also supported by the recent study of Janius et al. In a large study of 109 patients with CTD, NMO IgG seropositivity was restricted to those with NMO, LETM, or relapsing optic neuritis. The high syndrome specificity of NMO IgG in those patients suggests that this antibody is linked to the pathogenesis of NMO SD and argues against being part of polyclonal B-cell activation [15].

Patients with one autoimmune disease are predisposed to developing another autoimmune disease [16]. Our patient had increased ANA titer. It is reported that patients with NMO-SD may have positive autoimmune indices. ANA and SSA antibodies are detected in patients with NMO-SD who do not fulfill diagnostic criteria of a systemic autoimmune disease [17]. 

On the other hand, the presence of NMO IgG is considered as an index for the manifestation of an NMO spectrum disorder and predicts relapse and development of definite NMO [18]. It has been reported that coexistence of NMO with a systemic organ- or non-organ-specific autoimmune disorder or autoantibodies may be predictors of a poorer prognosis [18]. The current standard for patients with lupus and serious neurological complications is the administration of i.v. cyclophosphamide [19]. It is recently proposed that B-cell targeted therapy in the form of rituximab should be considered in patients with NMO and NMO-SD [20]. First- and second-line therapy schemes for NMO and NMO SD have been recently suggested by the task force. Azathioprine (2.5–3 mg daily) combined with oral prednisolone and rituximab is considered as first-line therapy while cyclophosphamide, mitoxantrone, and mycophenolate mofetil are second line. However, it is not clear whether NMOSD associated with autoimmune diseases requires different treatment strategies. Furthermore, for rituximab, the optimal surrogate measures and treatment intervals are unclear [21].

It is essential for clinicians to consider NMO-SD and to initiate aggressive immunosuppression to control severe relapses of myelitis. Even if a systemic autoimmune disease is suspected, as in our case, NMO spectrum disorders should be included in differential diagnosis and NMO-IgG antibodies should be tested. The detection of such antibodies in patients with known LTM represents a reliable marker for future severe relapse [18]. Yanagawa et al. reported that early detection of NMO-IgG combined with proper immunotherapy could represents the key to a good, long-term prognosis for limited forms of NMO [22]. 

Patients with LETM should always be tested for NMO IgG even if clinical and paraclinical autoimmune indices are present since patients with NMO-SD may present features of coexisting systemic autoimmunity. Long-term immunosuppressive treatment should be initiated as soon as the diagnosis is set, to prevent the attacks. Thorough followup for NMOSD patients not fulfilling criteria for CTD is mandatory to prevent severe relapses and ensure early diagnosis of a systemic autoimmune disease. Large studies of patients with NMO spectrum disorders may indicate the optimal drug regimen and treatment duration to attain complete relapse and recovery.
